# Symptomatic Postoperative Pneumocephalus: A Case Series and Review of Management Strategies

**DOI:** 10.1055/a-2710-4422

**Published:** 2025-10-08

**Authors:** Chris Marcellino, Christopher Koo

**Affiliations:** 1 Department of Anesthesiology and Perioperative Medicine, Mayo Clinic, Rochester, Minnesota, United States; 2 Department of Neurologic Surgery, Mayo Clinic, Rochester, Minnesota, United States

**Keywords:** pneumocephalus, nitrous oxide, postoperative complications, craniocerebral trauma, skull, cerebrospinal fluid leak

## Abstract

Postoperative (or postprocedural) pneumocephalus is unique from those associated with head injury, spontaneous cerebrospinal fluid leaks, and intracranial infection. Postoperative cranial imaging usually demonstrates a small volume of air that remains in the surgical bed, which is essentially self-limited and resolves over several weeks or less. However, occasionally, surgical defects lead to symptomatic postoperative air entrapment, and severe cases are generally due to one-way valves created by tissue, a mechanism shared with severe traumatic pneumocephalus. In the case where this causes progressive pressurization, this is termed tension pneumocephalus, analogous to its pulmonary counterpart. In the closed adult cranium, the Monroe-Kellie doctrine can be extended to include pneumocephalus if the compressible nature of gas is accounted for. Three illustrative cases are used to highlight common etiologies of postoperative tension pneumocephalus, management strategies, and imaging findings of these collections.

## Introduction

Usually, the postoperative sterile pneumocephalus is easily distinguishable on axial imaging and clinical history from those associated with head trauma, spontaneous cerebrospinal fluid (CSF) leaks, and intracranial infection with gas-producing species, 1 2 3 4 though at times combinations of these etiologies can contribute to challenging clinical circumstances. Postoperative cranial imaging generally demonstrates one or more non-compressive localized volumes of air that remain in the surgical bed, which are essentially self-limited and resolve over several weeks or less. Unless a mass removed was large or the brain had been chronically compressed (such as in a subdural hematoma), this collection is small and is often localized to the subdural and/or extradural space, depending on the dural closure technique used.

Resections performed in a dependent position, such as in sitting or prone posterior fossa neurosurgery in the reverse Trendelenburg position, can allow CSF to drain out of the cerebral convexities with a diffuse distribution of air in the basal cisterns, cerebral sulci and fissures, and occasionally the ventricles. The imaging results can be striking; however, the collections are generally benign. This air takes the place of the CSF, which had been drained from the surgical field and is generally low in total volume and non-compressive. In any approach, in the event of removal of large masses or large compressive lesions, such as a subdural hematoma, the residual air that fills the surgical void can be substantial if care is not taken to fill the surgical cavity with surgical irrigation. In these cases, excessive hyperventilation intraoperatively, particularly at the time of closure, can lead to collections under mild pressure. Hydrostatic forces ensure these are rapidly absorbed into the circulation, with typically transient brain compression of low concern.

On the other hand, in rare cases, pneumocephalus can develop or progress after a surgical wound has been closed. This is generally associated with dural injuries, which create one-way “ball-valves” in surrounding tissue (or with the use of positive pressure ventilation acutely after endonasal skull base surgery or other surgical violations of the anterior skull base 5 6 7 8 ). In the event that the air collection is progressively pressurized beyond ambient pressure, the term pressure or tension pneumocephalus is used, analogously to its use in tension pneumothorax. This portends a potentially progressive and more morbid condition.

The incidence and duration of postoperative and tension pneumocephalus have been studied. In a study of 240 patients who underwent craniotomy or craniectomy, as expected, 100% of patients had some degree of pneumocephalus on computed tomography (CT) imaging, regardless of size or symptomatology, which was, in most cases, asymptomatic. 9 There was a 25% resolution of postoperative pneumocephalus within the first week. At 2 weeks, 40.4% resolution occurred, and at 3 weeks, 73.6% resolution occurred. In another case study of 54 patients with all causes of otogenic pneumocephalus, tension pneumocephalus was present in 66% of cases, with trauma (36%) being the most common etiologic factor, while otitis media (30%), otologic surgery (30%), and congenital defects (2%) accounted for the remainder. 10

Generally, all patients with tension pneumocephalus or a persistent CSF leak will require revision surgery to repair the dural violation and potentially the underlying cause of the dural or surgical closure defect, and at times can have a protracted clinical course. Three illustrative case reports demonstrate the breadth of techniques required in the surgical and critical care management of these findings, with each demonstrating unique challenges that both surgeons and intensivists should be prepared for.

## Case Series

### Case 1

#### History and Examination

**Fig. 1:**
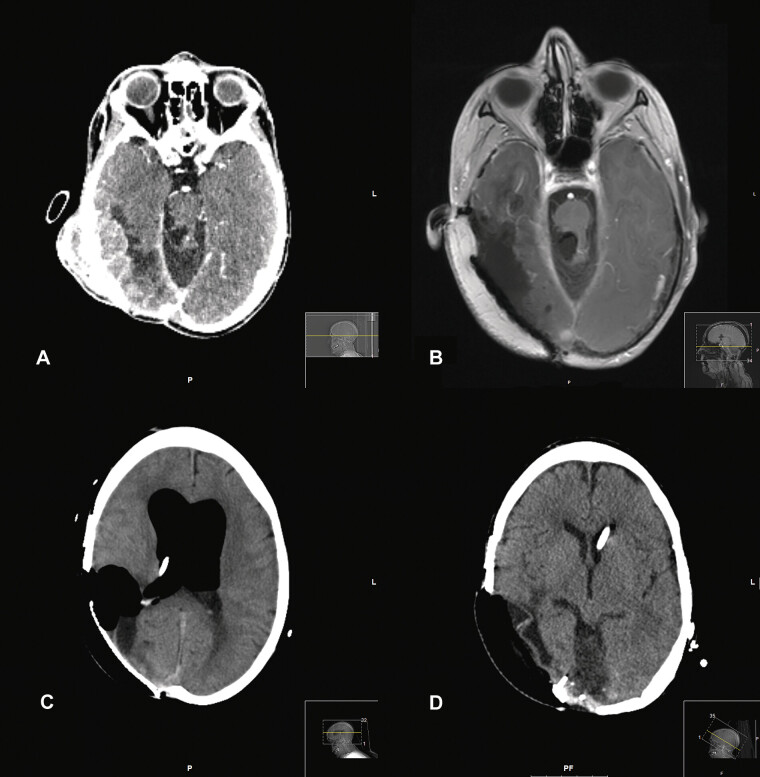
( **A** ) Axial image slice from a CT head study (with contrast) of findings of a radiation-associated meningioma in a 42-year-old man. This slice demonstrates extensive meningeal thickening, enhancement, and nodularity in a 7-cm diameter portion of the right temporoparietal convexity, suggestive of a recurrent meningioma. ( **B** ) Axial image slice from MRI brain study (contrast T1) postoperatively, 31 days later, showing postoperative changes without residual enhancing nodular soft tissue, suggestive of gross total resection of the meningioma. ( **C** ) Axial image slice from CT head study, an additional 9 days later, showing new multicompartment pneumocephalus in bilateral ventricles, the right posterior fossa postoperative cavity, right subdural spaces, and extension to the epidural space, as well as suprasellar cistern air and new hydrocephalus. ( **D** ) Axial image slice from CT head, an additional 4 days later, showing resolution of pneumocephalus, improvement of hydrocephalus, and new postoperative changes of closure of the right external auditory canal and a tympanomastoid obliteration with removal of the existing large cranioplasty mesh and removal and replacement of the free flap. CT, computed tomography.

A 42-year-old man had a history of childhood cerebellar astrocytoma that had been resected and treated with radiation. He subsequently developed hydrocephalus, requiring placement of a ventriculoperitoneal (VP) shunt. During adulthood, he developed a right temporoparietal meningioma, which recurred despite multiple resections and Gamma Knife radiosurgery. During the admission of interest, he presented with progressive hearing loss and regrowth of the meningioma ( Fig. 1A ).

#### Operation and Perioperative Course

He underwent resection of this meningioma via a retrolabyrinthine approach and a wide temporoparietal craniotomy, scalp resection and reconstruction with a vastus lateralis flap, and shunt externalization ( Fig. 1B ). Postoperatively, he developed right CSF otorrhea, which was thought to be originating from a small laceration of the right external auditory canal. The otorrhea persisted despite maximal medical management. Ten days after the procedure, his mental status declined, and repeat imaging demonstrated ventriculomegaly due to intraventricular pneumocephalus ( Fig. 1C ). The patient was intubated, a lumbar drain was placed, and a blind closure of the right external auditory canal and a tympanomastoid obliteration was performed with the removal of the existing large cranioplasty mesh and removal and replacement of the free flap ( Fig. 1D ). Later, he underwent internalization of his VP shunt and was discharged to rehabilitation.

### Case 2

#### History and Examination

**Fig. 2:**
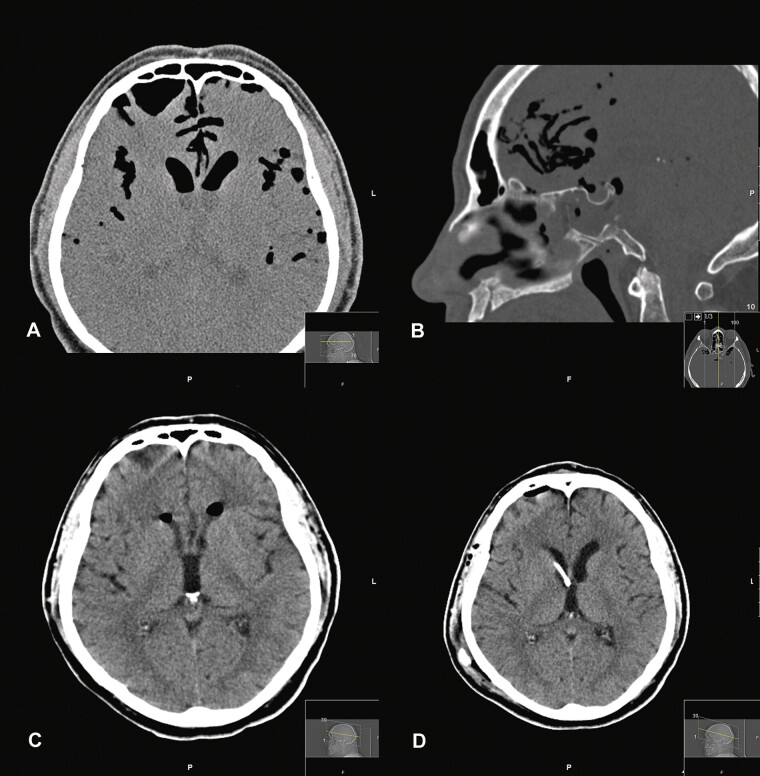
( **A, B** ) Axial and sagittal slices, respectively, from a CT sinus study of a 65-year-old man who underwent transnasal endoscopic resection of a large clival meningioma with subsequent placement of a fat graft and multilayered closure. He unfortunately expectorated his fat graft and developed CSF rhinorrhea and severe headache. These slices show postoperative material in the sphenoid sinus and along the posterior wall of the clivus in the area of the resected tumor, as well as a large amount of air in the Sylvian fissures and ventricles. ( **C** ) Axial image slice from CT head study 3 days later showing near resolution of cisternal air, with a small amount of air remaining in the Sylvian fissures and frontal horns of the lateral ventricles. ( **D** ) Axial image slice from a CT head study, an additional 10 days later, showing interval right frontal ventricular catheter placement. CSF, cerebrospinal fluid; CT, computed tomography.

A 65-year-old man who underwent transnasal endoscopic resection of a large clival meningioma with subsequent placement of an abdominal fat graft, Surgicel (oxidized regenerated cellulose; Ethicon, Inc., Raritan, NJ), Duragen (porous collagen matrix; Integra LifeSciences, Princeton, NJ), and then DuraSeal (polyethylene glycol hydrogel, Ethicon, Inc.) were used to seal the large defect at his skull base. However, after discharge at home, he expectorated the graft. He presented the following day with CSF rhinorrhea and a severe headache, but no focal neurological findings. A CT of his head showed significant free air intracranially along the frontal lobes, Sylvian fissures, and in the lateral ventricles ( Fig. 2A, B ).

#### Operation and Perioperative Course

He was maintained on cyclic oxygen therapy, which resulted in considerable improvement of the pneumocephalus ( Fig. 2C ). Later, a lumbar drain was placed, and his skull base defect was repaired with a new fat graft placement and bioabsorbable plating done via a revision transsphenoidal approach. (While the technique of plating is now considered controversial due to the increased risk of infection and the difficulty of revision surgery, it remains an infrequently used option in refractory cases. 11 ) Postoperatively, his course was complicated by a concern of infection, for which he completed a course of empiric antibiotics. He was unable to wean from the lumbar drain without leak recurrence; thus, a VP shunt was placed ( Fig. 2D ). One week after shunt placement, he was discharged home.

### Case 3

#### History and Examination

**Fig. 3:**
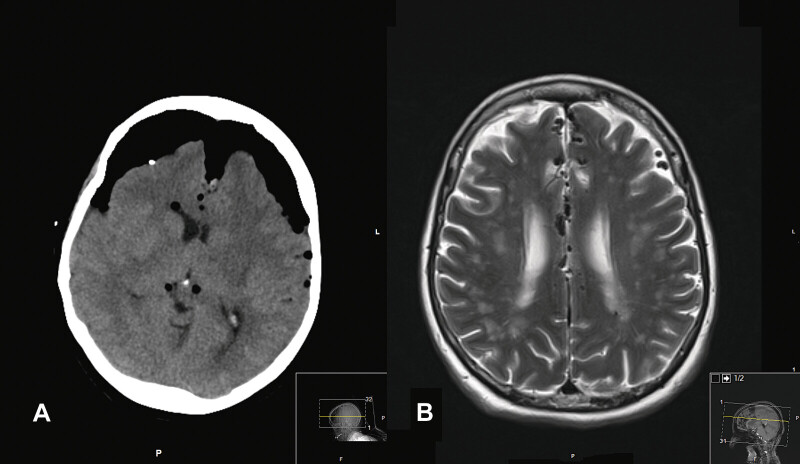
( **A** ) Axial image slice from preoperative MRI brain study (T2 sequence) showing scattered pneumocephalus along the falx and left frontal lobe, without significant mass effect in a 75-year-old female with a spontaneous left sphenoid CSF leak and meningitis. ( **B** ) Axial image slice from a postoperative CT head study, 16 days later, showing a large amount of intracranial air exerting a mass effect on the frontal and temporal lobes bilaterally. This is the classic “Mt. Fuji” sign of massive tension pneumocephalus. CSF, cerebrospinal fluid; CT, computed tomography.

A 75-year-old female had a history of spontaneous left sphenoid CSF leak and meningitis. This leak was initially repaired endoscopically with a nasoseptal flap 12 ( Fig. 3A ). However, 1 week after discharge, she began to experience severe headaches with altered mental status. Imaging performed suggested tension pneumocephalus and flap failure ( Fig. 3B ).

#### Operation and Perioperative Course

A burr hole was placed emergently at the bedside in the ICU using a cranial access kit (commonly used with bedside ventricular drain placement), and a revision flap reconstruction was performed in the operating room. Postoperatively, her mental status did not improve despite empirical antibiotic treatment for suspected meningitis. A repeat CT scan redemonstrated worsening pneumocephalus. Given this recurrent leak after a prolonged period, it was believed that intracranial hypertension was causing breakdown of her closure and most likely caused her original leak. She underwent a second repair of the nasoseptal flap with lumbar drain placement. Postoperative imaging initially demonstrated decreasing pneumocephalus; however, she later again developed recurrent CSF leak a third time, and symptomatic pneumocephalus. She returned to the operating room for a third repair and placement of a VP shunt, as it was suspected that the LP shunt likely failed. She was ultimately discharged on long-term antibiotics for suspected meningitis, but with resolution of the CSF leak and pneumocephalus.

## Discussion

The Monroe-Kellie doctrine states that the sum of the volumes of the brain, CSF, and blood within the adult cranial cavity is nearly 13 constant, and that a decrease in one necessitates an increase in one or more of the other two, and vice versa. 14 15 16 17 In certain pathological cases, this compartment accommodates a fourth: Gas. The physical distribution of gas within the cranial vault depends on the buoyant physics, as air is less dense than fluid and hence brain matter. For example, this results in the characteristic “Mt. Fuji” sign in the case of a tension pneumocephalus with the air shifted anteriorly and superiorly in the supine patient (see Fig. 3A ). 18

The vast majority 9 of open intracranial procedures result in a small residual volume of air being entrapped inside the surgical cavity, but these collections generally resolve spontaneously and rarely lead to persistent clinical symptoms unless large. In the postoperative patient, in addition to this air left in situ at the time of surgery, residual ambient–CSF communication or resultant or preexisting fistulae can let air entrain (or certain anesthetic gases by subsequent exchange) in any of the meningeal layers or inside the brain parenchyma or within the ventricles. 18 19 20 21 Increased risk in the sitting position for more extensive pneumocephalus is described, although the magnitude and clinical significance of this is probably low. 22 23 24 The majority of clinically significant or symptomatic pneumocephalus cases are due to temporal region or transnasal skull base operations that lead to open communication with the atmosphere and intracranial space (though it is the authors' opinion that the incidence of symptomatic pneumocephalus has greatly decreased in the era of routine placement of nasoseptal flaps 12 25 in anterior skull base reconstruction). Pneumocephalus after large-volume lumbar puncture drainage in patients with elevated pressures or spinal anesthesia has also been described. 26 27 Rare cases of symptomatic pneumocephalus have been described after VP shunt placement secondary to an insidious congenital otic 28 or sinus defect. 29

Symptoms of clinically significant pneumocephalus are primarily due to the mass effect imposed by the gas on the brain matter, including headaches, nausea, vomiting, altered sensorium, focal neurological deficits, seizures, and, in severe cases, coma and death secondary to herniation from a large gas collection in tension pneumocephalus. The clinical presentation is similar to other causes of elevated ICP or non-communicating hydrocephalus. Small collections of sterile pneumocephalus are generally asymptomatic, although air in the cranium can cause mild neurological symptoms even when not under pressure, such as lethargy, confusion, and headache (though these non-specific symptoms are often seen postcraniotomy in the absence of significant air). Untreated, these symptoms generally resolve in 1 to 3 days as the bulk of the air is resorbed into the circulation, though complete resolution may take weeks.

This diagnosis is made easily on CT imaging by identifying voxels of a region with Hounsfield units of −1,000 (see Figs. 1C , 2A , and 3B ) in the epidural and subdural spaces. Moderate and large collections are also easily seen on cranial plain films due to the lucency and sharp borders created by the air–tissue interface, though this is essentially a historical footnote at this point. Pneumocephalus can also enter the subarachnoid space if introduced iatrogenically, with cases describing cranial spread from air inadvertently introduced in the spine. 30 After evacuation of chronic subdural hematoma or resection of a large tumor, a large benign pneumocephalus may be difficult to distinguish from a tension pneumocephalus radiographically if the brain has yet to re-expand to occupy the residual intracranial space. 18 20 31 In any case, with comparison to preoperative images, the presence of a midline shift or imaging signs of herniation indicates the potential need for emergent treatment.

Historically, the use of nitrous oxide (N _2_ O) after the beginning of surgical closure was also associated with tension pneumocephalus due to the high solubility of nitrous oxide in the absence of ball-valve physiology. This can lead to a pressurized pneumocephalus due to the increased pressure caused by the equimolar exchange of nitrous oxide for the nitrogen gas in air, as the gas will enter gas-filled spaces at least 30 times faster than nitrogen gas can exit. 32 Cases have been described of mass effect from nitrous oxide use in the sitting position, even with discontinuation of nitrous oxide prior to wound closure, as the volatile gas exchanges for air that is trapped in the cerebral convexities from CSF that had drained out. This is a unique set of circumstances that can create life-threatening tension physiology even without continued air entrainment or a one-way tissue valve. 33 34 These collections are ultimately self-limited once administration of the gas is ceased (as nitrous oxide is rapidly cleared after its administration is stopped), but injuries have been described due to the transient mass effect, which can be substantial.

New foci of intracranial air can be seen postoperatively either due to deep surgical site infection or from contiguous infection from the nasal sinuses or mastoid, though these are seen in a distinct imaging pattern and time course postoperatively. Infectious causes of pneumocephalus are analogous to gas gangrene in other organ systems and are generally due to *Staphylococcus* sp., *Clostridium* sp., and anaerobic *Streptococcus* sp., and are often due to traumatic or iatrogenic inoculation. 4 Other gas-producing organisms can rarely cause pneumocephalus, including *Klebsiella* sp. in a diabetic. Rarely, neoplasms, especially osteomas, epidermoid tumors, and pituitary tumors, are associated with penetration of the dura with CSF leaks into the sinuses, Eustachian tubes, or external auditory canal, including delayed presentations associated with radiotherapy after resection. 35 36 37 38 39

## Management

Small collections of residual air without continued air entrapment or tension physiology can then be observed. If there is sufficient clinical concern or if the lesion is suspected to be symptomatic, bed rest and high concentrations of inspired oxygen for up to 24 hours 40 41 and serial imaging. With high concentrations of supplemental oxygen, the displacement of nitrogen by oxygen in the blood leads to an increased gradient of nitrogen in the pneumocephalus as compared with circulation, which increases the net rate of dissolution approximately two-fold due to mass action as well as intralesional exchange with the more soluble oxygen. 40 Due to the pulmonary toxicity 41 of high inspired concentrations of oxygen, treatment should be suspended after 24 hours and only repeated, or “cycled” as necessary thereafter, after a sufficient hiatus.

A finding of a collection larger than the surgical void or one that increases progressively in size should be concerning for a complex lesion where air is being continuously trapped into the wound by a one-way ball-valve formed by tissue or layers of dura or closure material. If imaging after an acute change in mental status or development of a focal deficit shows a large expanding collection of gas, this can be emergently temporized at the bedside by twist-drill evacuation and optionally placement of a ventricular catheter into the space (as opposed to the ventricle itself), open to a collection system or bag, or percutaneous aspiration can be performed through an existing cranial defect in the operative wound if possible. Emergent surgery can be performed to evacuate the pneumocephalus when indicated and to correct the underlying defect. In the case of recent transnasal skull base or sinus surgery, or middle fossa surgery, early intubation may be useful to reduce pharyngeal air pressure and the Venturi effect caused by ventilation past the defect. It may also be required if the mental status of the patient is sufficiently depressed or there is concern about airway protection. Infectious causes must be ruled out if suggested by laboratory or imaging findings, or clinical history, though large collections would be uncommon. When intracranial infection is a diagnostic possibility and is a potential cause of the gas collection, the need for source control of the infection must be determined, and appropriate targeted or empirical antibiotic therapy instituted.

If sudden deterioration and imaging findings of a large pneumocephalus are associated with the recent placement of a lumbar or external ventricular drain, consider clamping the drain, as it could be providing negative pressure, which could be drawing fluid past a one-way valve through an iatrogenic or congenital dural defect. 26 This is only pertinent under clinical circumstances where use of the drain appeared to trigger the clinical decline, as a functioning drain may be instrumental in controlling ICP and successful CSF leak repair in other cases.

The use of adjunctive closure materials such as harvested abdominal fat, oxidized cellulose polymer, and collagen or bovine pericardial dural patches has also been shown to be helpful in closing large dural defects, and in refractory cases, the use of these materials in repeated layers appears to be beneficial. Diversion of CSF with lumbar drains may aid in closure and healing of the leak site, and is almost always indicated in cases of intracranial hypertension 42 and multiply recurrent CSF leak or recurrent symptomatic pneumocephalus (though note that recent evidence 43 44 45 suggest little value and potential harm from a prophylactic placement of lumbar drains in transnasal neurosurgery in general). Acetazolamide can also be used for weeks to months after repair of a leak when there is suspicion of idiopathic intracranial hypertension (IIH) 46 47 to allow healing prior to confirmatory diagnosis of IIH, and a role for this agent has been established for first-line treatment of this condition in combination with weight loss. 48 49 Spontaneous CSF leaks have the highest recurrence rate of any leak, 50 and attention to sound closure (with a pedicled tissue flap 51 when possible) and management of intracranial hypertension if present is essential for sustained results. Broad-spectrum antibiotic prophylaxis should be considered and is often used routinely after postoperative CSF leaks, though evidence of benefit is inconclusive in promptly repaired leaks. 52 53 54 55

**Fig. 4:**
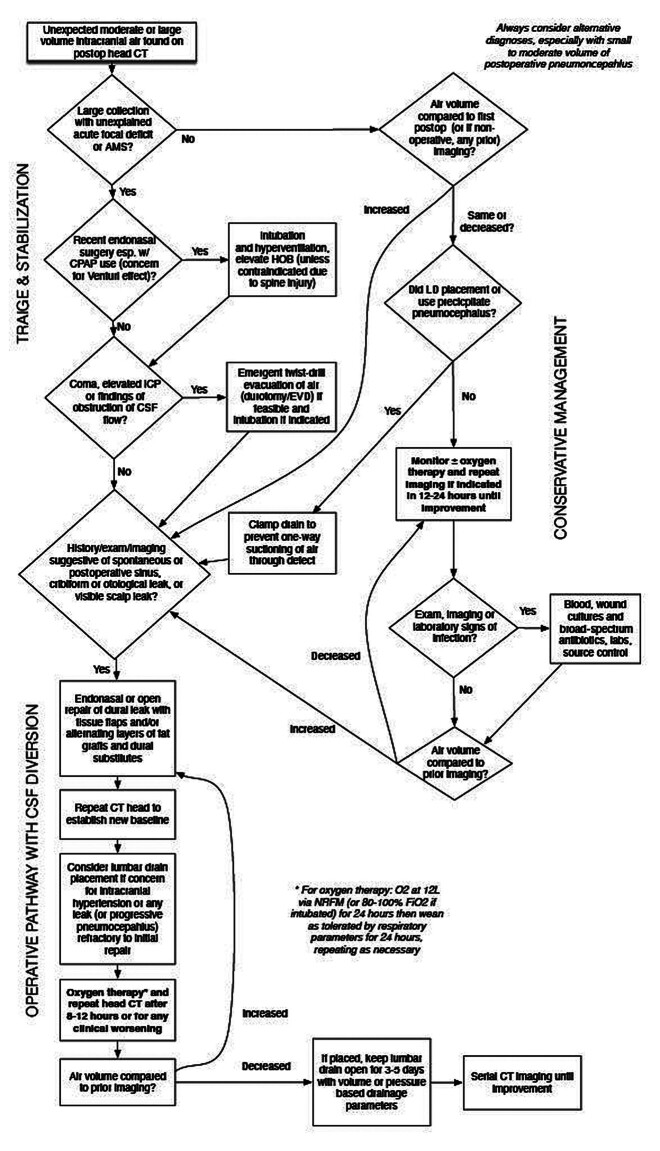
A suggested algorithm for the management of pneumocephalus. See the associated discussion in the “Management” section of the text. CT, computed tomography.

Though definitive guidelines do not exist, non-invasive positive pressure ventilation should be held in the immediate period after transnasal surgery or any neurosurgery where the anterior skull base is violated. The experience at our institution is that a period of withholding continuous positive airway pressure (CPAP) for 7 days is sufficient to minimize the risk of CSF leak and pneumocephalus when a vascularized pedicled flap is used, such as a nasoseptal flap, in wound closure. Other authors have drawn similar conclusions. 7 8 Figure 4 summarizes the management considerations and approaches discussed here.

## Conclusion

Most intracranial air observed on postoperative imaging is expected; however, when the amount of air is disproportionate to the wound bed, increases in volume, or becomes symptomatic, emergent intervention may be necessary. Managing complex or tension cases can often require multiple revision surgeries or procedures and a multidisciplinary approach to minimize the risk of significant morbidity. Fortunately, advancements in surgical techniques have significantly reduced the incidence of severe or symptomatic cases of this complication.
